# Supplementary material for: Ultrafast carbon monoxide photolysis and heme spin-crossover in myoglobin via nonadiabatic quantum dynamics
Source: Nat Commun. 2018 Oct 29;9:4502. doi: 10.1038/s41467-018-06615-1 (PMC6206034; doi:10.1038/s41467-018-06615-1)
Supplement: Supplementary file 1 — Supplementary information [file 41467_2018_6615_MOESM1_ESM.pdf]

# Ultrafast carbon monoxide photolysis and heme spin-crossover in myoglobin via nonadiabatic quantum dynamics

Falahati et al.

| State | Label | ANO-RCC-VDZ | Mixed basis set | Absolute Difference |
|---|---|---|---|---|
| 1 | $^5$MC | 0.70 | 0.82 | 0.12 |
| 2 | $^5$MC | 0.74 | 0.86 | 0.12 |
| 3 | $^3$MC | 0.89 | 0.92 | 0.02 |
| 4 | $^5$MC | 1.40 | 1.41 | 0.01 |
| 5 | $^3$MC | 1.43 | 1.43 | 0.00 |
| 6 | $^3$MC | 1.51 | 1.44 | 0.06 |
| 7 | $^3$MC | 1.62 | 1.69 | 0.06 |
| 8 | $^3$MC | 1.86 | 1.84 | 0.01 |
| 9 | $^3$MC | 1.90 | 1.89 | 0.01 |
| 10 | $^1$MC | 1.90 | 2.00 | 0.10 |
| 11 | $^3$ML | 2.15 | 2.34 | 0.19 |
| 12 | $^1$MC | 2.35 | 2.43 | 0.09 |
| 13 | $^1$MC | 2.36 | 2.45 | 0.08 |
| 14 | $^3Q_1$ | 2.43 | 2.46 | 0.03 |
| 15 | $^5$ML | 2.48 | 2.66 | 0.18 |
| 16 | $^3Q_2$ | 2.49 | 2.47 | 0.02 |
| 17 | $^3$ML | 2.56 | 2.57 | 0.01 |
| 18 | $^1Q_x$ | 2.73 | 2.71 | 0.02 |
| 19 | $^1Q_y$ | 2.79 | 2.71 | 0.07 |
| 20 | $^1$ML | 2.83 | 2.93 | 0.10 |

**Supplementary Table 1. Basis set effect.** Basis set size effect of the absorption spectrum of Hb-CO using ANO-RCC-VDZ basis set and a mixed basis set consisting of a ANO-RCC-VTZP for Iron with 6s5p3d contractions, ANO-RCC-VDZP for nitrogens and the carbon monoxide and a ANO-RCC-VDZ for the rest of the atoms.

| State | Label | Full virtual CASPT2 | Frozen virtual CASPT2 | Absolute Difference |
|---|---|---|---|---|
| 1 | $^5$MC | 0.70 | 0.42 | 0.28 |
| 2 | $^5$MC | 0.74 | 0.46 | 0.28 |
| 3 | $^3$MC | 0.89 | 0.79 | 0.10 |
| 4 | $^5$MC | 1.40 | 1.00 | 0.40 |
| 5 | $^3$MC | 1.43 | 1.23 | 0.20 |
| 6 | $^3$MC | 1.51 | 1.25 | 0.27 |
| 7 | $^3$MC | 1.62 | 1.58 | 0.04 |
| 8 | $^3$MC | 1.86 | 1.65 | 0.21 |
| 9 | $^3$MC | 1.90 | 1.72 | 0.18 |
| 10 | $^1$MC | 1.90 | 1.99 | -0.09 |
| 11 | $^3$ML | 2.15 | 1.77 | 0.38 |
| 12 | $^1$MC | 2.35 | 2.35 | 0.00 |
| 13 | $^1$MC | 2.36 | 2.38 | -0.02 |
| 14 | $^3Q_1$ | 2.43 | 1.96 | 0.47 |
| 15 | $^5$ML | 2.48 | 2.19 | 0.29 |
| 16 | $^3Q_2$ | 2.49 | 2.32 | 0.17 |
| 17 | $^3$ML | 2.56 | 2.43 | 0.13 |
| 18 | $^1Q_x$ | 2.73 | 2.79 | 0.06 |
| 19 | $^1Q_y$ | 2.79 | 2.85 | -0.06 |
| 20 | $^1$ML | 2.83 | 2.64 | 0.19 |

**Supplementary Table 2. Benchmark of CASPT2.** Comparison of CASPT2 using the full virtual space and CASPT2 freezing 116 virtual orbitals. In both cases, 37 core orbitals are frozen. The computational cost of CASPT2 calculations in the full space of orbitals is too demanding for practical uses. Therefore, we reduced the space of configurations in the perturbative treatment by freezing the core orbitals and 116 highest virtual orbitals.

| State | Energy (eV) | Osc. Strength | Interpretation | Label | Symmetry |
|---|---|---|---|---|---|
| 1 | 0.70 | 0.000 | $(d_{yz},d_{xy}) \rightarrow (d_{x^2-y^2},d_{z^2})$ | $^5$MC | E |
| 2 | 0.74 | 0.000 | $(d_{xz},d_{xy}) \rightarrow (d_{x^2-y^2},d_{z^2})$ | $^5$MC | E |
| 3 | 0.89 | 0.000 | $d_{xy} \rightarrow d_{x^2-y^2}$ | $^3$MC | $B_2$ |
| 4 | 1.40 | 0.000 | $(d_{xz},d_{yz}) \rightarrow (d_{x^2-y^2},d_{z^2})$ | $^5$MC | |
| 5 | 1.43 | 0.000 | $d_{xz} \rightarrow d_{z^2}$ | $^3$MC | E |
| 6 | 1.51 | 0.000 | $d_{yz} \rightarrow d_{z^2}$ | $^3$MC | E |
| 7 | 1.62 | 0.000 | $d_{xy} \rightarrow d_{z^2}$ | $^3$MC | $A_2$ |
| 8 | 1.86 | 0.000 | $d_{yz} \rightarrow d_{x^2-y^2}$ | $^3$MC | E |
| 9 | 1.90 | 0.000 | $d_{xz} \rightarrow d_{x^2-y^2}$ | $^3$MC | E |
| 10 | 1.90 | 0.000 | $d_{xy} \rightarrow d_{x^2-y^2}$ | $^1$MC | $B_2$ |
| 11 | 2.15 | 0.000 | $(a_2,d_{yz},d_{xy}) \rightarrow (e,d_{x^2-y^2},d_{z^2})$ | $^3$ML | |
| 12 | 2.35 | 0.000 | $d_{yz} \rightarrow d_{z^2}$ | $^1$MC | E |
| 13 | 2.36 | 0.000 | $d_{xy} \rightarrow d_{z^2}$ | $^1$MC | E |
| 14 | 2.43 | 0.000 | $a_2 \rightarrow e$ | $^3Q_1$ | E |
| 15 | 2.48 | 0.000 | $(a_2,d_{yz},d_{xy}) \rightarrow (e,d_{x^2-y^2},d_{z^2})$ | $^5$ML | |
| 16 | 2.49 | 0.000 | $a_2 \rightarrow e$ | $^3Q_2$ | E |
| 17 | 2.56 | 0.000 | $(a_1,d_{yz},d_{xy}) \rightarrow (e,d_{x^2-y^2},d_{z^2})$ | $^3$ML | |
| 18 | 2.73 | 0.015 | $a_2 \rightarrow e$ / $a_1 \rightarrow e$ | $^1Q_x$ | E |
| 19 | 2.79 | 0.010 | $a_2 \rightarrow e$ / $a_1 \rightarrow e$ | $^1Q_y$ | E |
| 20 | 2.83 | 0.000 | $(a_2,d_{yz}) \rightarrow (e,d_{z^2})$ | $^1$ML | |

**Supplementary Table 3. Heme–CO Absorption spectrum.** Absorption spectrum of Heme–CO model calculated at the CASSCF(10,9)/MS-CASPT2/ANO-RCC-VDZ level. The transitions in the interpretation are represented according to orbital excitations from the ground state closed-shell configuration of the leading MS-CASPT2 configuration. The labels used are either metal-centred (MC), metal-ligand-transition (ML) and Q for the porphyrin states. The symmetry assignments are only approximate, considering that the distal hystidin has only a spectator role and the states can be assigned according to a $C_{4v}$ point group.

| State | Energy (eV) | Interpretation | Label |
|---|---|---|---|
| 1 | 0.65 | $(a_1,d_{xz},d_{yz})\rightarrow(d_{z^2},e,d_{x^2-y^2})$ | $^3$ML |
| 2 | 0.67 | $(a_1,d_{xz},d_{yz})\rightarrow(d_{z^2},e,d_{x^2-y^2})$ | $^5$ML |
| 3 | 0.72 | $(a_1,d_{xz},d_{xy})\rightarrow(d_{z^2},e,d_{x^2-y^2})$ | $^5$ML |
| 4 | 0.72 | $(a_1,d_{yz},d_{xy})\rightarrow(d_{z^2},e,d_{x^2-y^2})$ | $^3$ML |
| 5 | 0.72 | $(a_1,d_{yz},d_{xy})\rightarrow(d_{z^2},e,d_{x^2-y^2})$ | $^5$ML |
| 6 | 0.74 | $(a_1,d_{yz},d_{xy})\rightarrow(d_{z^2},e,d_{x^2-y^2})$ | $^3$ML |
| 7 | 0.76 | $(d_{xz},d_{xy})\rightarrow(d_{z^2},d_{x^2-y^2})$ | $^3$MC |
| 8 | 0.82 | $(a_1,d_{xz},d_{xy})\rightarrow(d_{z^2},e,d_{x^2-y^2})$ | $^5$ML |
| 9 | 0.83 | $(a_1,d_{xz},d_{yz})\rightarrow(d_{z^2},e,d_{x^2-y^2})$ | $^5$ML |
| 10 | 0.84 | $(a_1,d_{xy},d_{yz})\rightarrow(d_{z^2},e,d_{x^2-y^2})$ | $^5$ML |
| 11 | 0.85 | $(a_1,d_{xz},d_{xy})\rightarrow(d_{z^2},e,d_{x^2-y^2})$ | $^3$ML |
| 12 | 0.90 | $(d_{yz},d_{xy})\rightarrow(d_{z^2},d_{x^2-y^2})$ | $^3$MC |
| 13 | 0.91 | $(a_1,d_{xy},d_{yz})\rightarrow(d_{z^2},e,d_{x^2-y^2})$ | $^5$ML |
| 14 | 0.94 | $(a_2,d_{xy},d_{yz},d_{xz})\rightarrow(d_{z^2},d_{z^2},e,d_{x^2-y^2})$ | $^3$ML |
| 15 | 0.94 | $(a_2,d_{yz})\rightarrow(d_{z^2},e)$ | $^1$ML |
| 16 | 0.96 | $(a_1,d_{xy},d_{xz})\rightarrow(d_{z^2},e,d_{x^2-y^2})$ | $^5$ML |
| 17 | 0.99 | $(a_1,d_{xy},d_{xz})\rightarrow(d_{z^2},e,d_{x^2-y^2})$ | $^5$ML |
| 18 | 1.00 | $(a_2,d_{xy},d_{yz},d_{xz})\rightarrow(d_{z^2},d_{z^2},e,d_{x^2-y^2})$ | $^3$ML |
| 19 | 1.02 | $(d_{xy},d_{xz})\rightarrow(d_{z^2},d_{x^2-y^2})$ | $^3$MC |
| 20 | 1.07 | $(d_{xz},d_{yz})\rightarrow(d_{z^2},d_{x^2-y^2})$ | $^3$MC |
| 21 | 1.10 | $(d_{xz},d_{yz})\rightarrow(d_{z^2},d_{x^2-y^2})$ | $^3$MC |
| 22 | 1.15 | $(d_{xy},d_{yz})\rightarrow(d_{z^2},d_{x^2-y^2})$ | $^3$MC |
| 23 | 1.17 | $(a_2,d_{yz})\rightarrow(d_{z^2},e)$ | $^1$ML |
| 24 | 1.18 | $(d_{yz},d_{xz})\rightarrow(d_{z^2},d)$ | $^1$MC |

**Supplementary Table 4. Reduced model absorption spectrum.** Absorption spectrum of porphyrin-Fe-CO model (without the imidazole ring) calculated at the CASSCF(10,9)/MS-CASPT2/ANO-RCC-VDZ level. The transitions in the interpretation are represented according to orbital excitations from the ground state closed-shell configuration of the leading MS-CASPT2 configuration. The labels used are either metal-centred (MC), metal-ligand-transition (ML) and Q for the porphyrine states. The symmetry assignments are only approximate, considering that the distal hystidine has only a spectator role and the states can be assigned according to a $C_{4v}$ point group.

| State | Energy (eV) | Interpretation | Label |
|---|---|---|---|
| 1 | -1.76 | $(d_{xz},d_{xy})\rightarrow(d_{z^2},d_{x^2-y^2})$ | $^5$MC |
| 2 | -1.73 | $(d_{xz},d_{yz})\rightarrow(d_{z^2},d_{x^2-y^2})$ | $^5$MC |
| 3 | -1.09 | $(d_{xy},d_{yz})\rightarrow(d_{z^2},d_{x^2-y^2})$ | $^5$MC |
| 4 | -0.66 | $d_{xz} \rightarrow d_{z^2}$ | $^3$MC |
| 5 | -0.57 | $d_{yz} \rightarrow d_{z^2}$ | $^3$MC |
| 6 | -0.18 | $d_{xy} \rightarrow d_{z^2}$ | $^3$MC |
| 7 | -0.01 | $(d_{xz},d_{yz})\rightarrow(d_{x^2-y^2}, d_{x^2-y^2})$ | $^3$MC |
| 8 | 0.17 | $(a_2,d_{yz},d_{xz})\rightarrow(d_{z^2},e,d_{x^2-y^2})$ | $^3$ML |
| 9 | 0.18 | $(a_2,d_{yz},d_{xz})\rightarrow(d_{z^2},e,d_{x^2-y^2})$ | $^3$ML |
| 10 | 0.20 | $(a_2,d_{yz},d_{xy})\rightarrow(d_{z^2},e,d_{x^2-y^2})$ | $^3$ML |
| 11 | 0.20 | $(a_2,d_{yz},d_{xy})\rightarrow(d_{z^2},e,d_{x^2-y^2})$ | $^3$ML |
| 12 | 0.20 | $(d_{xy},d_{yz},d_{xz})\rightarrow(d_{z^2},d_{z^2},d_{x^2-y^2})$ | $^5$MC |
| 13 | 0.28 | $(d_{xy},d_{yz},d_{xz})\rightarrow(d_{z^2},d_{x^2-y^2},d_{x^2-y^2})$ | $^5$MC |
| 14 | 0.29 | $(a_2,d_{xy},d_{xz})\rightarrow(d_{z^2},e,d_{x^2-y^2})$ | $^5$ML |
| 15 | 0.32 | $(a_1,d_{yz},d_{xy})\rightarrow(d_{z^2},e,d_{x^2-y^2})$ | $^3$ML |
| 16 | 0.33 | $(a_2,d_{yz},d_{xz})\rightarrow(d_{z^2},e,d_{x^2-y^2})$ | $^5$ML |
| 17 | 0.35 | $(a_2,d_{xy},d_{xz})\rightarrow(d_{z^2},e,d_{x^2-y^2})$ | $^3$ML |
| 18 | 0.35 | $(a_2,d_{yz},d_{xz})\rightarrow(d_{z^2},e,d_{x^2-y^2})$ | $^5$ML |
| 19 | 0.36 | $(a_2,d_{xy},d_{xz})\rightarrow(d_{z^2},e,d_{x^2-y^2})$ | $^5$ML |
| 20 | 0.36 | $(a_2,d_{xy},d_{xz})\rightarrow(d_{z^2},e,d_{x^2-y^2})$ | $^3$ML |
| 21 | 0.46 | $(a_2,d_{yz},d_{xy})\rightarrow(d_{z^2},e,d_{x^2-y^2})$ | $^5$ML |
| 22 | 0.48 | $d_{yz} \rightarrow d_{z^2}$ | $^1$MC |
| 23 | 0.49 | $d_{xy} \rightarrow d_{x^2-y^2}$ | $^3$MC |
| 24 | 0.49 | $(a_2,d_{yz},d_{xy})\rightarrow(d_{z^2},e,d_{x^2-y^2})$ | $^5$ML |
| 25 | 0.52 | $(a_1,d_{yz},d_{xz})\rightarrow(d_{z^2},e,d_{x^2-y^2})$ | $^3$ML |
| 26 | 0.54 | $(a_1,d_{xz},d_{xy})\rightarrow(d_{z^2},e,d_{x^2-y^2})$ | $^5$ML |
| 27 | 0.57 | $d_{yz} \rightarrow d_{x^2-y^2}$ | $^3$MC |
| 28 | 0.58 | $d_{xz} \rightarrow d_{z^2}$ | $^1$MC |
| 29 | 0.59 | $d_{xz} \rightarrow d_{x^2-y^2}$ | $^3$MC |
| 30 | 0.63 | $(a_1,d_{xz},d_{yz})\rightarrow(d_{z^2},e,d_{x^2-y^2})$ | $^5$ML |

**Supplementary Table 5. Decarboxy model absorption spectrum.** Spectrum of the imidazole-Heme model calculated at the CASSCF(10,9)/MS-CASPT2/ANO-RCC-VDZ level. The ground state is now a quintet state, but the configurations are represented as excitations to the singlet closed shell configuration. The transitions in the interpretation are represented according to orbital excitations from the ground state closed-shell configuration of the leading MS-CASPT2 configuration. The labels used are either metal-centred (MC), metal-ligand-transition (ML) and Q for the porphyrine states.

| | Gas phase | | | QM/MM | | |
|---|---|---|---|---|---|---|
| State | Energy (eV) | Osc. Strength | Interpretation | Energy (eV) | Osc. Strength | Interpretation |
| 1 | 1.602 | 0.0000 | $d_{yz} \rightarrow e_x$ | 1.067 | 0.0002 | $d_{xy} \rightarrow d_{x^2-y^2}$ |
| 2 | 1.795 | 0.0000 | $d_{xz} \rightarrow e_y$ | 1.154 | 0.0011 | $d_{xy} \rightarrow e_y$ |
| 3 | 1.825 | 0.0001 | $(d_{xz}, \pi) \rightarrow (e_y, d_{x^2-y^2})$ | 1.188 | 0.0012 | $d_{xz} \rightarrow e_x$ |
| 4 | 2.547 | 0.0001 | $\pi \rightarrow e_y$ | 1.257 | 0.0012 | $d_{yz} \rightarrow e_x$ |
| 5 | 2.604 | 0.0000 | $(d_{yz}, \pi) \rightarrow (e_x, d_{x^2-y^2})$ | 1.353 | 0.0008 | $d_{yz} \rightarrow d_{z^2}$ |
| 6 | 2.805 | 0.0000 | $(d_{xz}, \pi) \rightarrow (e_y, d_{x^2-y^2})$ | 1.729 | 0.0001 | $d_{xy} \rightarrow d_{z^2}$ |
| 7 | 2.858 | 0.0000 | $(d_{xz}, \pi) \rightarrow (e_x, d_{x^2-y^2})$ | 2.062 | 0.0000 | $d_{yz} \rightarrow e_y$ |
| 8 | 2.919 | 0.0001 | $(d_{yz}, \pi) \rightarrow (e_x, d_{z^2})$ | 2.094 | 0.0001 | $d_{yz} \rightarrow e_x$ |
| 9 | 3.036 | 0.0001 | $d_{yz} \rightarrow e_y$ | 2.137 | 0.0001 | $d_{yz} \rightarrow e_x$ |
| 10 | 3.063 | 0.0000 | $d_{xz} \rightarrow e_x$ | 2.350 | 0.0000 | $(d_{xy}, \pi) \rightarrow (e_x, d_{x^2-y^2})$ |
| 11 | 3.074 | 0.0000 | $\pi \rightarrow e_x$ | 2.423 | 0.0002 | $(d_{xy}, \pi) \rightarrow (e_y, d_{x^2-y^2})$ |

**Supplementary Table 6. Effect of the protein on the absorption spectrum.** Comparison of the singlet manifold absorption spectrum of full Heme–CO calculated in the gas phase and at the QM/MM level employing the CASSCF(10,9)/MS-CASPT2/ANO-RCC-VDZ method. The transitions in the interpretation are represented according to orbital excitations from the ground state closed-shell configuration of the leading MS-CASPT2 configuration.

| State | CT | MC | LC | MLCT | LMCT |
|---|---|---|---|---|---|
| 1 | 8.4 | 91.5 | 0.1 | 7.7 | 0.7 |
| 2 | 13.1 | 86.5 | 0.4 | 9.7 | 3.4 |
| 3 | 12.8 | 86.8 | 0.4 | 9.6 | 3.2 |
| 4 | 80.5 | 3.7 | 15.9 | 9.3 | 71.2 |
| 5 | 0.8 | 0.0 | 99.2 | 0.1 | 0.8 |
| 6 | 0.8 | 0.0 | 99.2 | 0.1 | 0.7 |
| 7 | 72.2 | 18.9 | 9.0 | 71.1 | 1.1 |
| 8 | 52.7 | 34.8 | 12.5 | 51.3 | 1.4 |
| 9 | 84.9 | 2.0 | 13.1 | 2.4 | 82.6 |
| 10 | 85.6 | 3.3 | 11.0 | 6.5 | 79.1 |
| 11 | 12.7 | 87.0 | 0.3 | 11.6 | 1.1 |
| 12 | 18.9 | 79.4 | 1.7 | 13.8 | 5.1 |
| 13 | 13.2 | 86.3 | 0.5 | 11.7 | 1.4 |
| 14 | 11.7 | 88.0 | 0.3 | 8.1 | 3.6 |
| 15 | 11.7 | 88.0 | 0.3 | 8.3 | 3.4 |
| 16 | 11.9 | 87.6 | 0.5 | 8.5 | 3.4 |
| 17 | 20.7 | 74.6 | 4.7 | 16.9 | 3.8 |
| 18 | 91.7 | 0.4 | 8.0 | 0.2 | 91.4 |
| 19 | 38.4 | 61.1 | 0.4 | 36.2 | 2.2 |
| 20 | 55.1 | 44.4 | 0.5 | 53.5 | 1.6 |
| 21 | 87.8 | 3.9 | 8.3 | 19.4 | 68.4 |
| 22 | 76.2 | 2.3 | 21.5 | 11.6 | 64.6 |
| 23 | 86.7 | 5.1 | 8.3 | 22.0 | 64.7 |
| 24 | 9.5 | 90.0 | 0.5 | 4.9 | 4.6 |
| 25 | 37.0 | 60.7 | 2.2 | 32.4 | 4.6 |
| 26 | 69.3 | 29.7 | 1.1 | 67.8 | 1.5 |
| 27 | 91.9 | 0.4 | 7.7 | 1.6 | 90.3 |
| 28 | 83.7 | 5.6 | 10.7 | 10.7 | 73.0 |
| 29 | 83.0 | 5.5 | 11.6 | 35.9 | 47.1 |
| 30 | 11.3 | 88.4 | 0.3 | 8.2 | 3.1 |
| 31 | 11.3 | 88.4 | 0.3 | 8.1 | 3.3 |
| 32 | 69.2 | 25.8 | 5.0 | 43.1 | 26.1 |
| 33 | 12.9 | 86.1 | 1.0 | 4.3 | 8.6 |
| 34 | 11.4 | 88.5 | 0.1 | 10.5 | 0.9 |

**Supplementary Table 7. State character analysis.** Analysis of singlet one-particle transition density matrices between S0 and an excited state. The descriptors shown are the percentage of charge transfer (CT), metal-centered (MC), ligand-centered (LC), metal-to-ligand charge-transfer (MLCT) and ligand-to-metal charge-transfer (LMCT). Here, the ligand is considered as the porphyrin, the imidazole and the carbon monoxide. Calculations have been performed with TheoDore [http://theodore-qc.sourceforge.net].

| Dynamics: Mode | 1 | 2 | 3 | 4 | 5 | 6 | 7 | 8 | 9 | 10 |
|---|---|---|---|---|---|---|---|---|---|---|
| 8 | 0.0000 | 2.2086 | 1.5679 | 1.6863 | 2.5719 | 0.9910 | 1.2120 | 0.9525 | 1.5832 | 0.9146 |
| 12 | 0.0000 | -0.4742 | -0.4310 | -0.8618 | -0.1105 | -0.4513 | 0.4665 | -0.8952 | 0.1435 | -0.8237 |
| 16 | 0.0000 | -0.5391 | -0.3823 | 0.4011 | -0.9246 | -0.5033 | -0.7360 | -0.4054 | -0.4926 | 0.1645 |
| 17 | 0.0000 | -0.3425 | -0.0658 | -0.2078 | 0.2837 | 0.7531 | 0.1011 | 0.3937 | 0.1641 | 0.6317 |
| 18 | 0.0000 | -0.1482 | 0.2775 | 0.1946 | 0.5238 | -0.1025 | 0.0816 | 0.4920 | 0.7240 | -0.3165 |
| 21 | 0.0000 | -0.7377 | -0.5452 | 0.4596 | -0.2184 | 0.2309 | 0.4338 | -0.4165 | -0.4131 | -0.6170 |
| 22 | 0.0000 | 0.1569 | -0.3785 | 0.5238 | 0.1211 | 0.3889 | -0.6952 | 0.1619 | -0.4442 | 0.5767 |
| 23 | 0.0000 | -1.0742 | -1.1350 | -0.6131 | -0.5799 | -0.5441 | 0.1255 | -0.1479 | 0.1814 | -0.6622 |
| 26 | 0.0000 | -0.2251 | 0.5508 | 0.1869 | -0.4410 | -0.0811 | -0.0355 | 0.3209 | 0.0182 | -0.0112 |
| 27 | 0.0000 | -0.2665 | 0.0038 | -0.6745 | 0.2467 | -0.0803 | -0.1198 | -0.6621 | -0.3058 | -0.1000 |
| 28 | 0.0000 | 0.0648 | 0.2926 | -0.0249 | 0.4756 | 0.3370 | -0.6968 | 0.4235 | -0.0985 | -0.1914 |
| 33 | 0.0000 | -1.0949 | -1.2167 | -0.8405 | -0.8761 | -1.0008 | -0.5934 | -0.5653 | -0.8813 | -1.1415 |
| 35 | 0.0000 | 0.4457 | 0.2481 | 0.2740 | 0.1896 | 0.0275 | -0.1423 | 0.0850 | -0.0243 | -0.0763 |
| 36 | 0.0000 | 0.1459 | 0.0943 | -0.4371 | -0.4038 | -0.3701 | -0.2683 | -0.3734 | -0.0788 | -0.0327 |
| 122 | 0.0000 | 0.0046 | 0.0032 | 0.0017 | 0.0068 | 0.0107 | 0.0012 | 0.0009 | 0.0027 | 0.0036 |

**Supplementary Table 8. Quantum dynamics initial conditions.** Set of 10 initial conditions extracted from the molecular dynamics simulation on *Equus caballus* myoglobin-CO complex.[1] The values correspond to the displacement of the corresponding normal mode. These have been obtained by extracting the cartesian coordinates from different MD snapshots and projecting them in the basis of normal modes used to parametrize the model Hamiltonian.

| | $\tau_{S-T}$ | $\tau_{T-Q}$ | $\tau_Q$ |
|---|---|---|---|
| 1 | 60.63 | 252.77 | 9.40 |
| 2 | 81.71 | 428.40 | 22.15 |
| 3 | 69.13 | 471.50 | 19.98 |
| 4 | 80.58 | 450.39 | 29.81 |
| 5 | 72.21 | 473.90 | 24.34 |
| 6 | 89.61 | 422.64 | 28.65 |
| 7 | 55.36 | 497.29 | 29.12 |
| 8 | 67.79 | 430.95 | 31.87 |
| 9 | 74.13 | 477.34 | 32.06 |
| 10 | 111.32 | 386.60 | 29.97 |

**Supplementary Table 9. Photodynamic timescales.** Time scales (in femtoseconds) for the reactions singlet→triplet ($\tau_{S-T}$), triplet→quintet ($\tau_{T-Q}$) and $^1$Q→$^1$MLCT ($\tau_Q$) reactions.

| | Hb-CO (S=0) | | Heme-CO (S=0) | | deoxyHb (S=2) | Experimental |
|---|---|---|---|---|---|---|
| Mode | Frequency ($cm^{-1}$) | Assignment | Irrep | Frequency ($cm^{-1}$) | Frequency ($cm^{-1}$) | |
| 1 | 23.968 | Histidine rotation | | | 24.050 | |
| 2 | 45.903 | Histidine tortion y | | | 27.573 | |
| 3 | 60.377 | Porphyrine OOP | $B_2$ | 50.556 | 61.344 | |
| 4 | 63.552 | Porphyrine OOP | $B_1$ | 51.072 | 76.511 | |
| 5 | 71.554 | CO torsion x | E | 73.124 | | |
| 6 | 75.489 | CO torsion y | E | 73.124 | | |
| 7 | 81.311 | Histidine torsion x + CO torsion x | | | 39.843 | |
| 8 | 90.431 | Porphyrine OOP + Fe OOP | $A_1$ | 103.024 | | |
| 9 | 159.815 | Porphyrine OOP | E | 155.859 | 154.110 | |
| 10 | 161.986 | Porphyrine OOP | E | 155.859 | | |
| 11 | 196.854 | Porphyrine + Histidine torsion y | | | 128.362 | |
| 12 | 208.513 | Histidine | | | 171.12 | |
| 13 | 213.640 | Histidine | | | 164.262 | |
| 14 | 215.485 | Porphyrine IP | $B_1$ | 224.988 | 169.725 | |
| 15 | 231.528 | Porphyrine IP | $B_2$ | 230.836 | 200.559 | |
| 16 | 263.807 | Fe IP + Porphyrine | E | 250.744 | 224.089 | |
| 17 | 264.796 | Fe IP + Porphyrine | E | 250.744 | 229.581 | |
| 18 | 286.811 | Porphyrine + Fe OOP + CO | $A_1$ | 261.785 | 240.334 | |
| 19 | 303.997 | Porphyrine OOP | $A_2$ | 297.757 | 314.693 | |
| 20 | 315.167 | Porphyrine OOP | $B_2$ | 290.045 | | |
| 21 | 318.540 | Histidine + Fe + CO | E | 294.998 | 252.307 | |
| 22 | 323.305 | Porphyrine IP + Histidine + Fe | E | 294.998 | 529.615 | |
| 23 | 356.220 | Porphyrine IP | $A_1$ | 355.681 | 351.828 | |
| 24 | 365.077 | Porphyrine IP | E | 368.402 | 390.730 | |
| 25 | 365.623 | Porphyrine IP | E | 368.402 | 390.455 | |
| 26 | 376.370 | Fe-CO | $A_1$ | 371.630 | 365.044 | |
| 27 | 407.592 | Porphyrine IP | E | 411.154 | 390.455 | |
| 28 | 409.115 | Porphyrine IP | E | 411.154 | | |
| 29 | 426.112 | Porphyrine IP | $B_1$ | 432.304 | 417.838 | |
| 30 | 428.517 | Porphyrine IP | $A_2$ | 431.203 | | |
| 31 | 452.146 | Porphyrine OOP | E | 452.146 | 459.769 | |
| 32 | 454.646 | Porphyrine OOP | E | 454.646 | 453.576 | |
| 33 | 488.045 | Fe OOP-CO dissociation | $A_1$ | 546.790 | 505 | Biophys. J. 80, 2372 (2001) |
| 34 | 498.135 | Porphyrine OOP | $B_1$ | 494.800 | 500.870 | |
| 35 | 574.101 | CO y | E | 512.680 | | |

**Supplementary Table 10. Vibrational modes.** Comparison of the vibrational modes of Hb-CO , Heme-CO and deoxyHb models in their lowest energy state. Modes are calculated at the B3LYP/LAND2Z level. The assignment is based on the Hb-CO vibrations, classified as out-of-plane (OOP) in-plane (IP) porphyrine distortions; and histidine or CO contributions if present. The vibrations of the Heme-CO are classified according to the irreducible representations of the $C_{4v}$ point group.

| | Hb-CO (S=0) | | Heme-CO (S=0) | | deoxyHb (S=2) | Experimental |
|---|---|---|---|---|---|---|
| Mode | Frequency ($cm^{-1}$) | Assignment | Irrep | Frequency ($cm^{-1}$) | Frequency ($cm^{-1}$) | |
| 36 | 578.414 | CO x | E | 512.680 | | |
| 37 | 627.151 | Histidine OOP | | | 628.629 | |
| 38 | 663.391 | Histidine OOP | E | 693.760 | 666.358 | |
| 39 | 690.059 | Histidine OOP | E | 693.760 | 683.881 | |
| 40 | 690.282 | Porphyrine OOP | $B_2$ | 689.033 | 689.358 | |
| 41 | 694.239 | Histidine - Porphyrine | E | 729.814 | 696.314 | |
| 42 | 694.826 | Porphyrine OOP | E | 729.814 | 694.491 | |
| 43 | 707.539 | Porphyrine OOP | $A_2$ | 707.080 | 709.262 | |
| 44 | 713.224 | Porphyrine OOP | $B_1$ | 711.495 | 712.473 | |
| 45 | 727.126 | Porphyrine OOP | E | 729.814 | 722.003 | |
| 46 | 727.296 | Porphyrine OOP | E | 729.814 | 722.951 | |
| 47 | 733.864 | Porphyrine OOP | $A_1$ | 735.822 | 729.998 | |
| 48 | 738.498 | Porphyrine IP | $A_1$ | 740.495 | 733.763 | |
| 49 | 751.022 | Porphyrine IP | $B_2$ | 751.596 | 749.306 | |
| 50 | 755.698 | Porphyrine IP | E | 757.182 | 754.127 | |
| 51 | 757.599 | Porphyrine IP | E | 757.182 | 751.727 | |
| 52 | 766.822 | Histidine | | | 771.125 | |
| 53 | 795.577 | Porphyrine OOP | $A_1$ | 802.791 | 795.159 | |
| 54 | 808.014 | Porphyrine OOP | E | 815.213 | 809.935 | |
| 55 | 808.947 | Porphyrine OOP | E | 815.213 | 806.644 | |
| 56 | 820.252 | Porphyrine IP | E | 820.252 | 817.218 | |
| 57 | 820.475 | Porphyrine OOP | $B_2$ | 826.982 | 821.368 | |
| 58 | 821.592 | Porphyrine IP | E | 821.592 | 817.216 | |
| 59 | 822.077 | Porphyrine IP | $A_2$ | 823.925 | 816.016 | |
| 60 | 839.722 | Porphyrine IP | $B_1$ | 840.489 | | |
| 61 | 864.232 | Histidine | | | 861.810 | |
| 62 | 894.401 | Porphyrine OOP | $B_1$ | 904.909 | 895.379 | |
| 63 | 903.468 | Porphyrine OOP | E | 914.968 | 902.507 | |
| 64 | 903.495 | Porphyrine OOP | E | 914.968 | 903.938 | |
| 65 | 911.925 | Histidine | | | 908.850 | |
| 66 | 912.551 | Porphyrine OOP | $A_1$ | 925.183 | 913.185 | |
| 67 | 940.174 | Histidine | | | 936.449 | |
| 68 | 945.844 | Porphyrine OOP | $A_2$ | 951.693 | 945.263 | |
| 69 | 950.152 | Porphyrine OOP | E | 957.084 | 949.281 | |
| 70 | 950.668 | Porphyrine OOP | E | 957.084 | 949.989 | |

Supplementary Table 10 continued.

| | Hb-CO (S=0) | | Heme-CO (S=0) | | deoxyHb (S=2) | Experimental |
|---|---|---|---|---|---|---|
| Mode | Frequency ($cm^{-1}$) | Assignment | Irrep | Frequency ($cm^{-1}$) | Frequency ($cm^{-1}$) | |
| 71 | 953.022 | Porphyrine OOP | $B_1$ | 959.950 | 952.690 | |
| 72 | 960.326 | Histidine | | | 955.155 | |
| 73 | 1004.994 | Porphyrine IP | $A_1$ | 1004.670 | 1010.627 | |
| 74 | 1007.752 | Porphyrine IP | E | 1006.664 | 997.052 | |
| 75 | 1008.293 | Porphyrine IP | E | 1006.664 | 997.969 | |
| 76 | 1012.775 | Porphyrine IP | $B_2$ | 1012.925 | 1016.082 | |
| 77 | 1017.522 | Porphyrine IP | $A_2$ | 1014.043 | 1011.281 | |
| 78 | 1045.157 | Porphyrine IP | E | 1044.314 | 1022.711 | |
| 79 | 1045.473 | Porphyrine IP | E | 1044.314 | 1028.178 | |
| 80 | 1066.686 | Porphyrine IP | $B_1$ | 1063.944 | 1041.765 | |
| 81 | 1079.060 | Histidine | | | 1071.447 | |
| 82 | 1093.939 | Porphyrine IP | E | 1098.789 | 1090.598 | |
| 83 | 1095.198 | Porphyrine IP | $B_2$ | 1099.920 | 1090.008 | |
| 84 | 1095.738 | Porphyrine IP | E | 1098.789 | 1092.252 | |
| 85 | 1100.602 | Porphyrine IP | $A_1$ | 1104.292 | 1097.432 | |
| 86 | 1120.780 | Histidine | | | 1100.953 | |
| 87 | 1153.970 | Histidine | | | 1149.020 | |
| 88 | 1176.726 | Porphyrine IP | $A_2$ | 1172.944 | 1178.186 | |
| 89 | 1183.063 | Porphyrine IP | E | 1182.383 | 1180.107 | |
| 90 | 1184.091 | Porphyrine IP | E | 1182.383 | 1177.492 | |
| 91 | 1185.583 | Histidine | | | 1178.377 | |
| 92 | 1210.142 | Porphyrine IP | $B_2$ | 1208.288 | 1193.620 | |
| 93 | 1226.794 | Porphyrine IP | $B_1$ | 1232.760 | 1227.271 | |
| 94 | 1281.238 | Porphyrine IP | E | 1290.151 | 1272.409 | |
| 95 | 1285.932 | Porphyrine IP | E | 1290.151 | 1269.135 | |
| 96 | 1286.115 | Histidine | | | 1282.184 | |
| 97 | 1336.933 | Porphyrine IP | E | 1348.808 | 1329.042 | |
| 98 | 1341.099 | Porphyrine IP | E | 1348.808 | 1324.720 | |
| 99 | 1358.770 | Histidine | | | 1356.220 | |
| 100 | 1364.077 | Porphyrine IP | $A_2$ | 1362.268 | 1357.120 | |
| 101 | 1280.021 | Porphyrine IP | $B_1$ | 1384.785 | 1372.126 | |
| 102 | 1382.059 | Porphyrine IP | $A_1$ | 1387.072 | 1363.478 | |
| 103 | 1386.884 | Porphyrine IP | $A_2$ | 1389.220 | 1386.338 | |
| 104 | 1423.294 | Porphyrine IP | E | 1414.903 | 1415.336 | |
| 105 | 1426.226 | Porphyrine IP | E | 1414.903 | 1419.005 | |
| 106 | 1428.835 | Porphyrine IP | $B_2$ | 1419.971 | 1417.301 | |
| 107 | 1435.426 | Histidine | | | 1424.590 | |
| 108 | 1479.803 | Porphyrine IP | E | 1494.741 | 1466.759 | |
| 109 | 1483.509 | Porphyrine IP | $A_1$ | 1494.379 | 1459.360 | |
| 110 | 1485.770 | Porphyrine IP | E | 1494.741 | 1459.156 | |

Supplementary Table 10 continued.

| Hb-CO (S=0) | | | Heme-CO (S=0) | | deoxyHb (S=2) | Experimental |
|---|---|---|---|---|---|---|
| Mode | Frequency ($cm^{-1}$) | Assignment | Irrep | Frequency ($cm^{-1}$) | Frequency ($cm^{-1}$) | |
| 111 | 1500.737 | Histidine | | | 1495.243 | |
| 112 | 1521.576 | Porphyrine IP | $B_1$ | 1535.518 | 1495.212 | |
| 113 | 1534.326 | Porphyrine IP | $B_2$ | 1534.326 | 1522.337 | |
| 114 | 1566.881 | Histidine | | | 1558.140 | |
| 115 | 1575.189 | Porphyrine IP | E | 1586.025 | 1552.244 | |
| 116 | 1578.558 | Porphyrine IP | E | 1586.025 | 1557.822 | |
| 117 | 1600.825 | Porphyrine IP | $A_1$ | 1611.892 | 1579.420 | |
| 118 | 1619.396 | Porphyrine IP | E | 1641.536 | 1589.724 | |
| 119 | 1629.202 | Porphyrine IP | E | 1641.539 | 1595.611 | |
| 120 | 1642.585 | Porphyrine IP | $A_2$ | 1656.540 | 1610.861 | |
| 121 | 1684.060 | Porphyrine IP | $B_2$ | 1707.299 | 1653.108 | |
| 122 | 1978.449 | CO | $A_1$ | 1971.316 | | |
| 123 | 3207.619 | Porphyrine | | H-wagging | | |
| 124 | 3207.893 | Porphyrine | | H-wagging | | |
| 125 | 3208.446 | Porphyrine | | H-wagging | | |
| 126 | 3208.955 | Porphyrine | | H-wagging | | |
| 127 | 3250.737 | Porphyrine | | H-wagging | | |
| 128 | 3250.849 | Porphyrine | | H-wagging | | |
| 129 | 3250.881 | Porphyrine | | H-wagging | | |
| 130 | 3251.577 | Porphyrine | | H-wagging | | |
| 131 | 3277.367 | Porphyrine | | H-wagging | | |
| 132 | 3277.826 | Porphyrine | | H-wagging | | |
| 133 | 3277.875 | Porphyrine | | H-wagging | | |
| 134 | 3278.256 | Porphyrine | | H-wagging | | |
| 135 | 3319.446 | Histidine | | H-wagging | | |
| 136 | 3347.201 | Histidine | | H-wagging | | |
| 137 | 3356.502 | Histidine | | H-wagging | | |
| 138 | 3690.449 | Histidine | | H-wagging | | |

Supplementary Table 10 continued.

| Mode 1 | Mode 2 | Mode 3 | $V_{i,j,k}$ ($cm^{-1}$) |
|---|---|---|---|
| 22 | 8 | 8 | -14.6863 |
| 22 | 16 | 16 | -11.7859 |
| 22 | 18 | 18 | -80.8503 |
| 22 | 21 | 21 | -77.1663 |
| 23 | 22 | 8 | -22.7241 |
| 23 | 22 | 21 | -15.1951 |
| 23 | 23 | 22 | -68.5081 |
| 26 | 22 | 8 | -38.1493 |
| 26 | 22 | 18 | -10.7142 |
| 26 | 26 | 22 | -115.6509 |
| 27 | 12 | 8 | -13.1565 |
| 27 | 16 | 16 | 14.9445 |
| 27 | 21 | 21 | -82.3505 |
| 27 | 23 | 12 | 16.8431 |
| 27 | 23 | 23 | 13.1509 |
| 27 | 26 | 12 | 29.1195 |
| 27 | 27 | 22 | -17.8932 |
| 28 | 26 | 26 | 11.9516 |
| 33 | 26 | 22 | -35.7878 |
| 33 | 33 | 22 | 11.3630 |
| 35 | 23 | 22 | 21.1882 |
| 35 | 27 | 21 | 18.4865 |
| 35 | 35 | 22 | 16.5150 |
| 36 | 22 | 12 | 33.5126 |
| 36 | 27 | 8 | -31.1431 |
| 122 | 122 | 17 | 35.9359 |

**Supplementary Table 11. Anharmonic vibrational couplings.** Third-order anharmonic mode couplings $V_{i,j,k}$ in $cm^{-1}$ in the space of normal modes used to expand our vibronic model Hamiltonian. Only couplings with absolute value larger than 10 $cm^{-1}$ are shown.

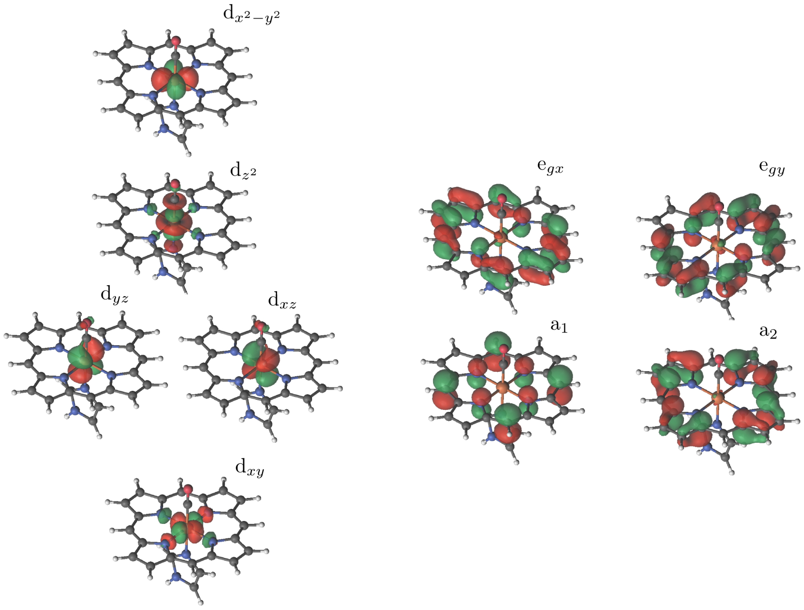


**Supplementary Figure 1. Active space orbitals.** Active space orbitals employed in the CASSCF(10,9)/CASPT2 calculations. Two occupied orbitals and two virtual orbitals correspond to the porphyrin moiety and two occupied and two virtuals for the Fe centre.

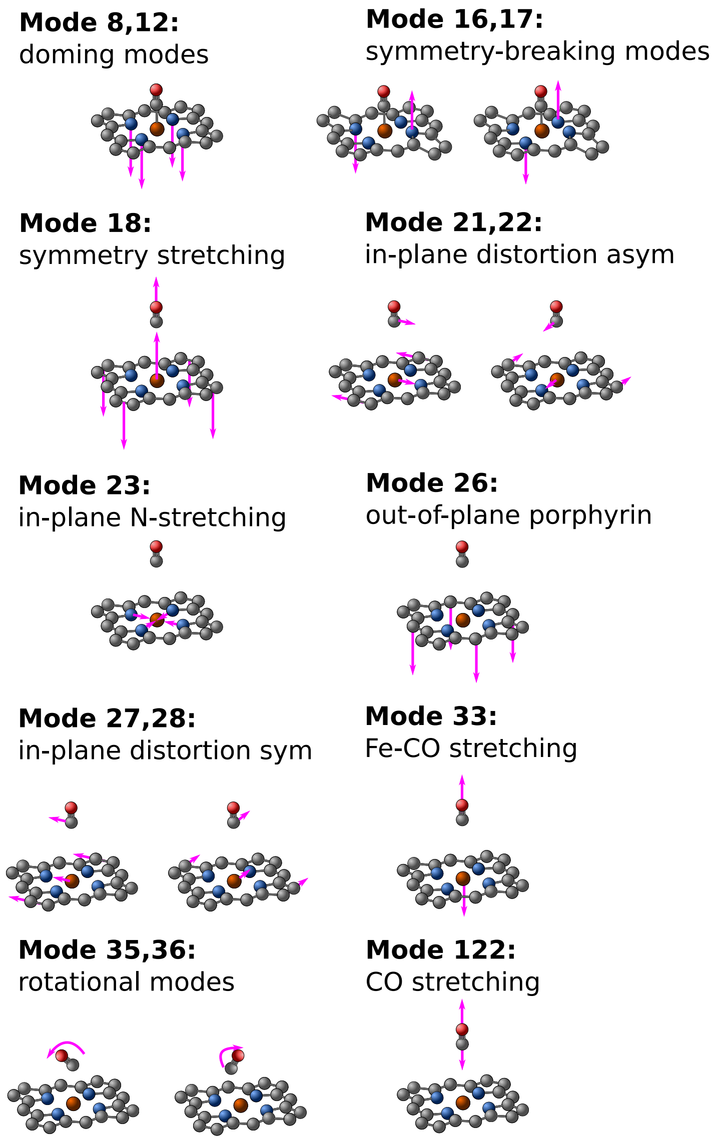


**Supplementary Figure 2. Normal modes in the vibronic model.** Schematic representation of the 15 vibrational modes included in the vibronic model with their interpretation. For a detailed analysis of all normal modes, see Supplementary Table 10.

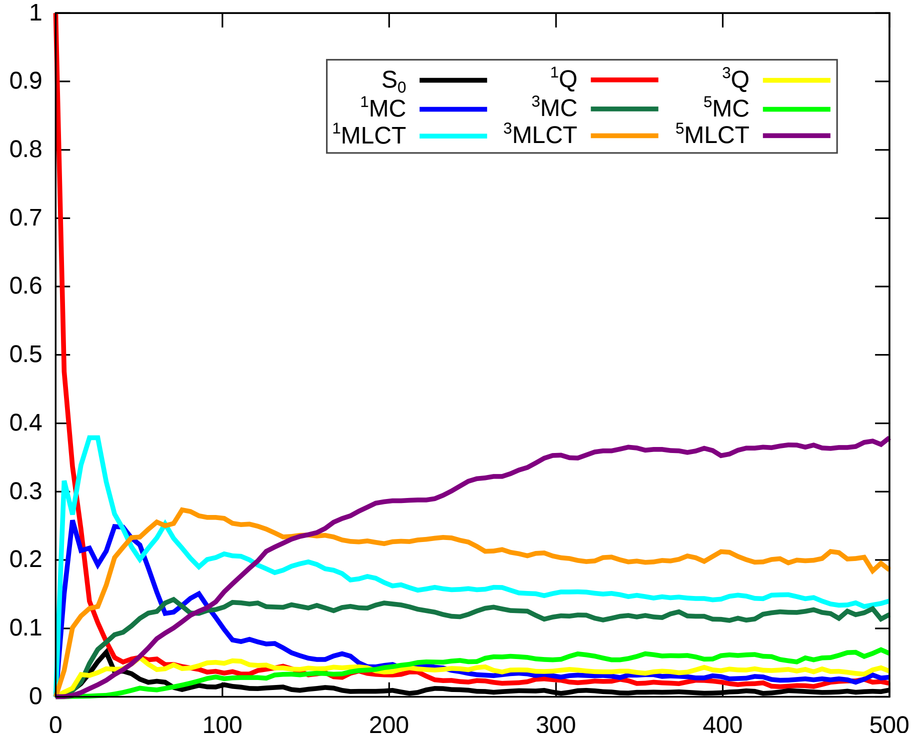


**Supplementary Figure 3. Detailed full model population evolution.** Evolution of diabatic populations in detail. The population evolution contains the ground state (S0), the singlet metal centred (dark blue), the singlet metal-ligand charge transfer band (light blue), the singlet Q state (red), the triplet metal centred (dark green), the triplet metal-ligand charge transfer (orange), the triplet Q state (yellow), the quintet metal centred state (light green) and the metal-ligand charge transfer (purple). Time (x-axis) is in femtoseconds.

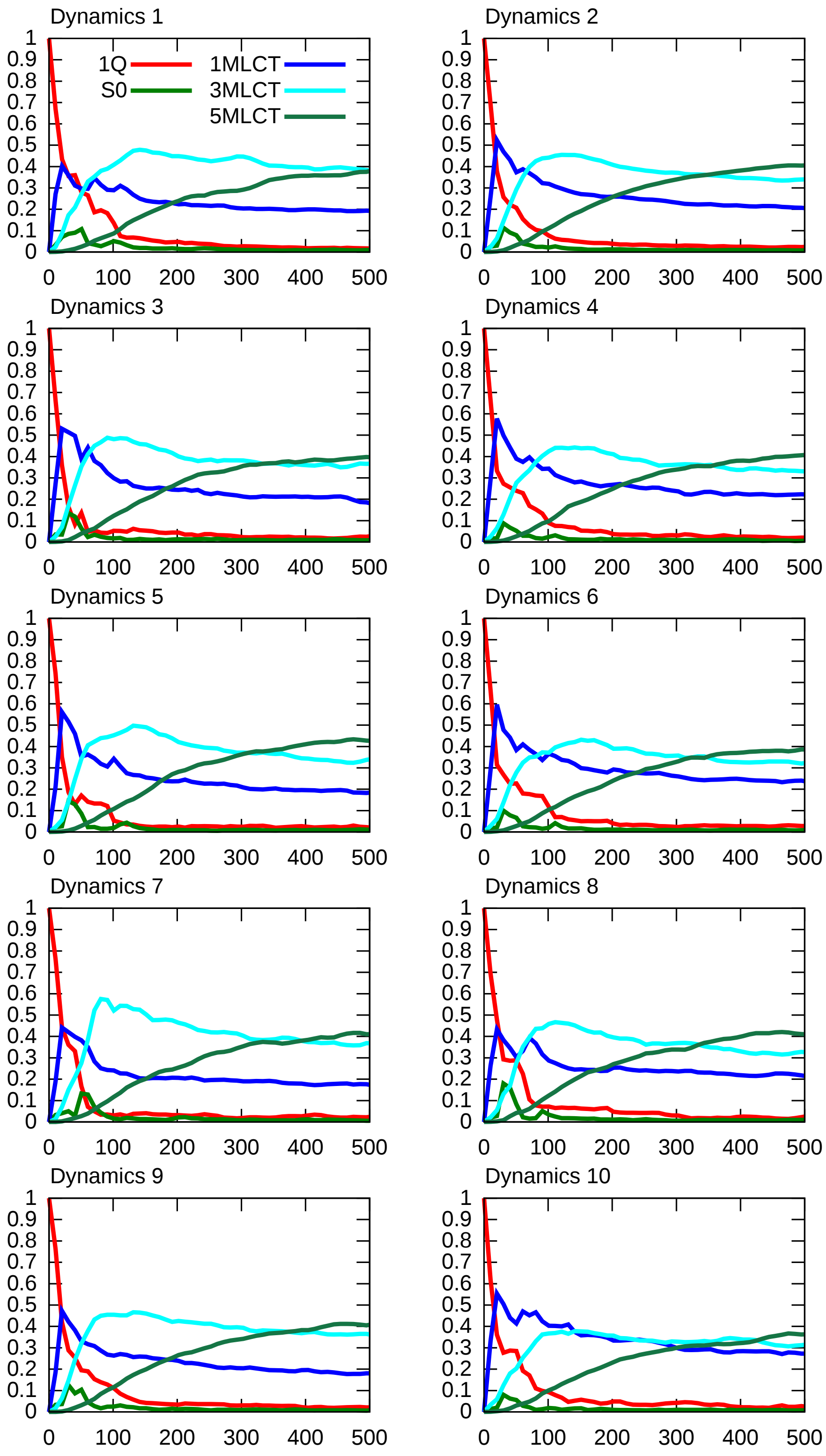


**Supplementary Figure 4. Full model population evolution.** Evolution of diabatic populations of the total singlet (red) triplet (green) and quintet (blue) manifold populations. Time (x-axis) is in femtoseconds.

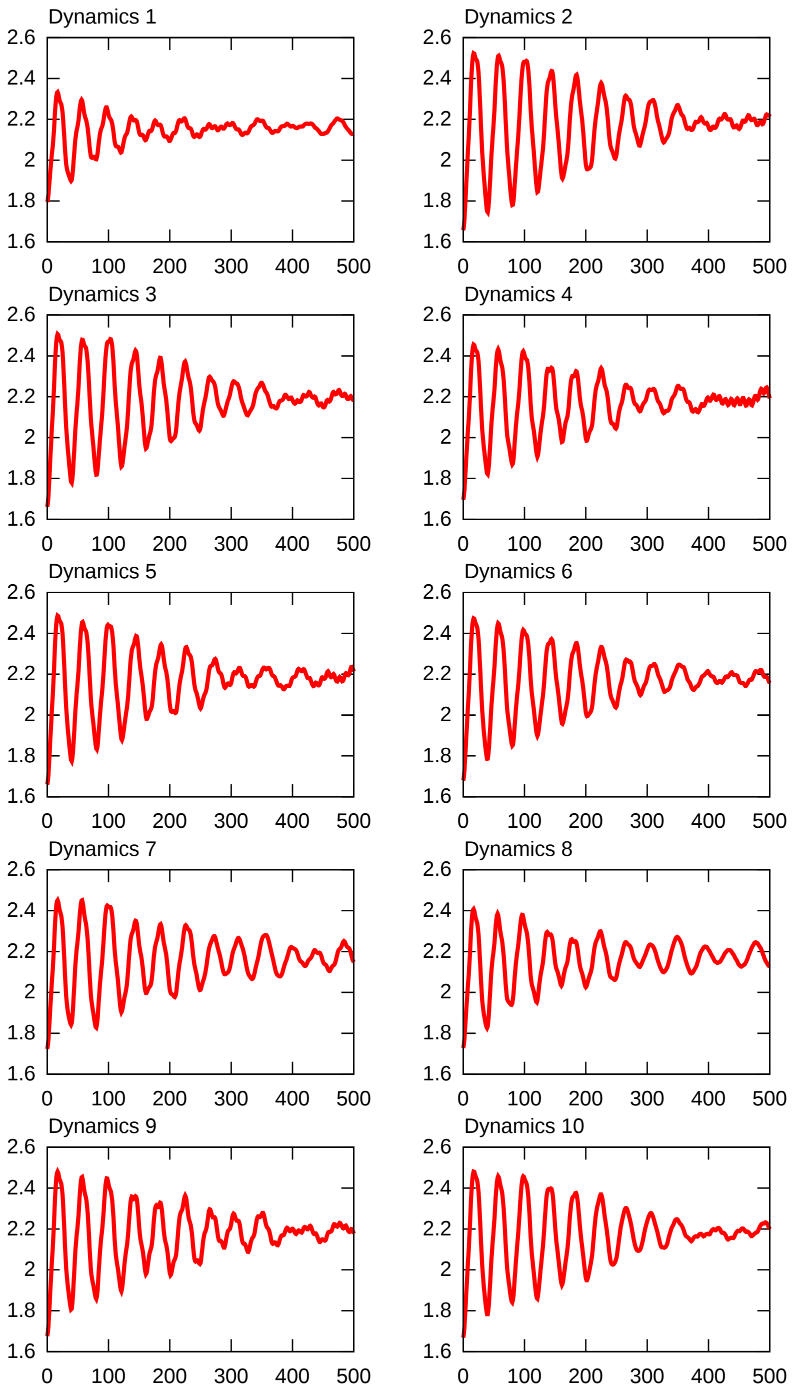


**Supplementary Figure 5. Evolution of the dissociation coordinate in the full model.** Evolution of the Fe-C(O) distance in Angstrom for the different initial conditions specified in Supplementary Table 8.

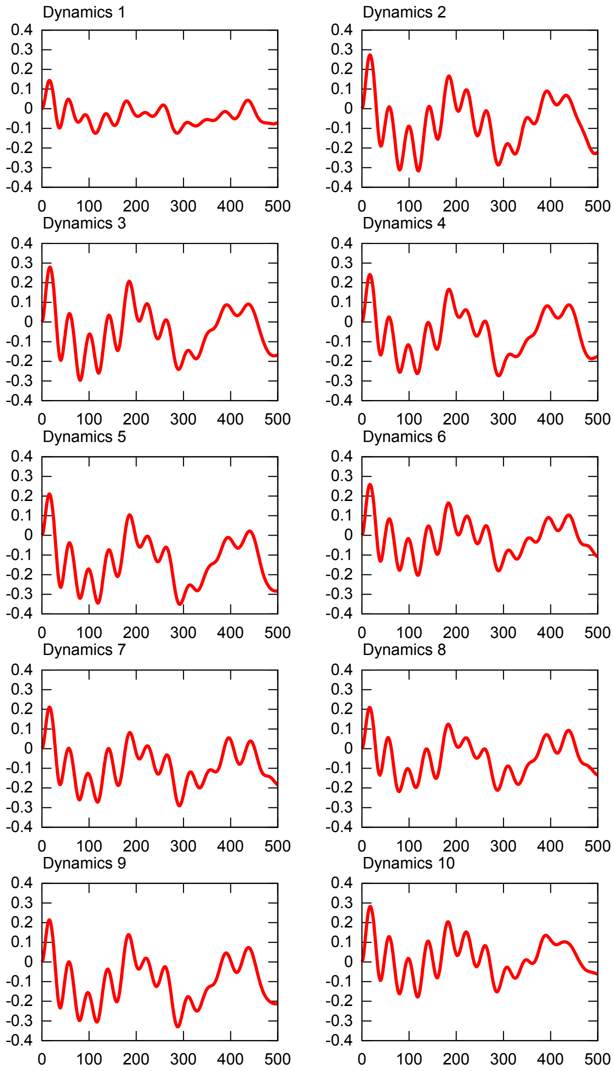


**Supplementary Figure 6. Evolution of the Fe OOOP coordinate in the full model.** Dynamic evolution of the Fe out-of-plane distance for the full model. The distance is calculated as the difference between the Fe z-coordinate position and the rest z-coordinate position (corresponding to the minimum energy geometry optimized at the B3LYP/LANL2DZ level). Positive and negative values of Fe indicates that Fe is going towards and contrary to the CO position respectively.

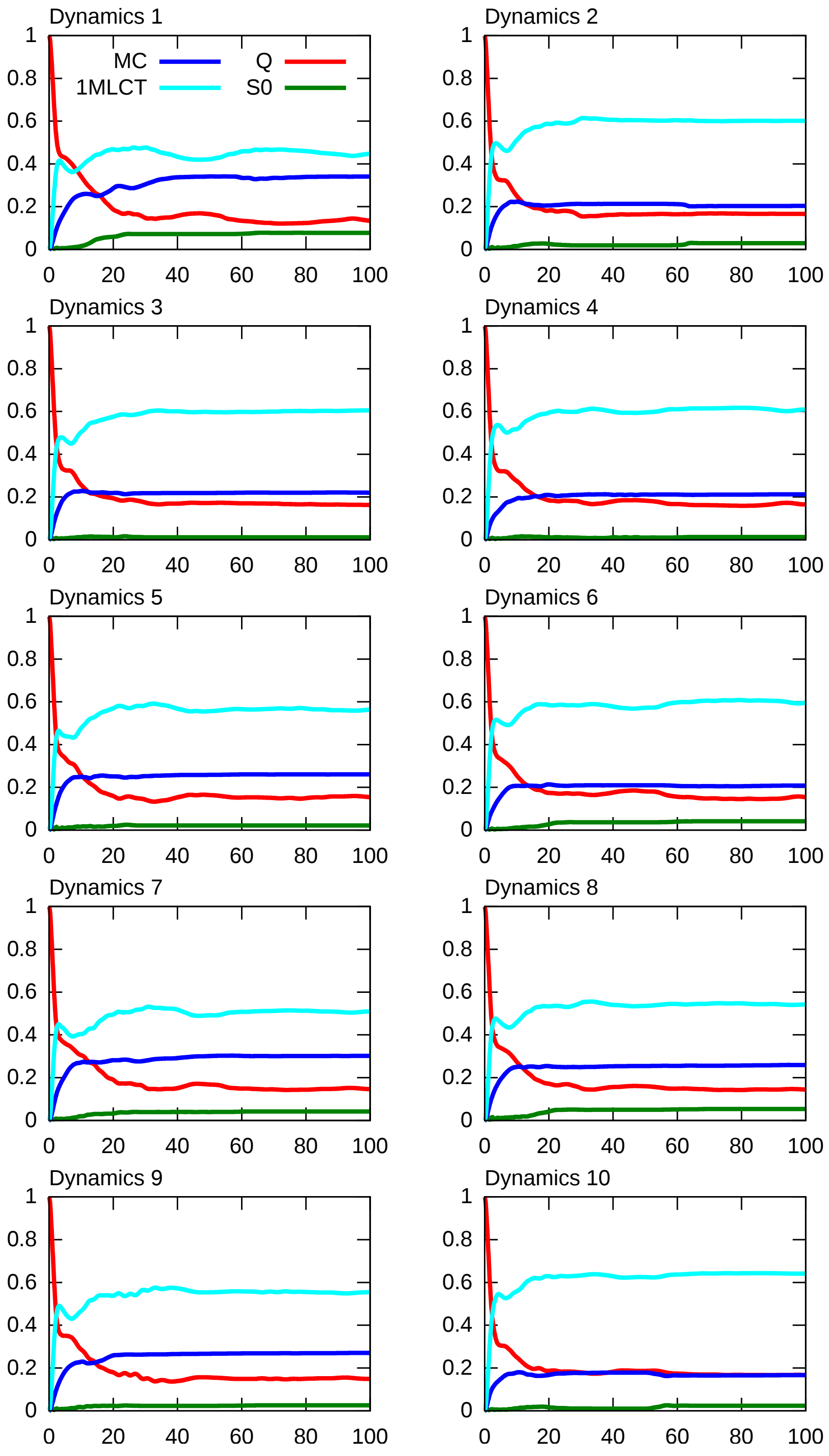


**Supplementary Figure 7. Singlet manifold population evolution.** Evolution of diabatic populations of the ground state $S_0$ (red), the summed-up populations of the metal-centred states (green), the Q-band (blue) and the MLCT band (cyan). Time (x-axis) is in femtoseconds.

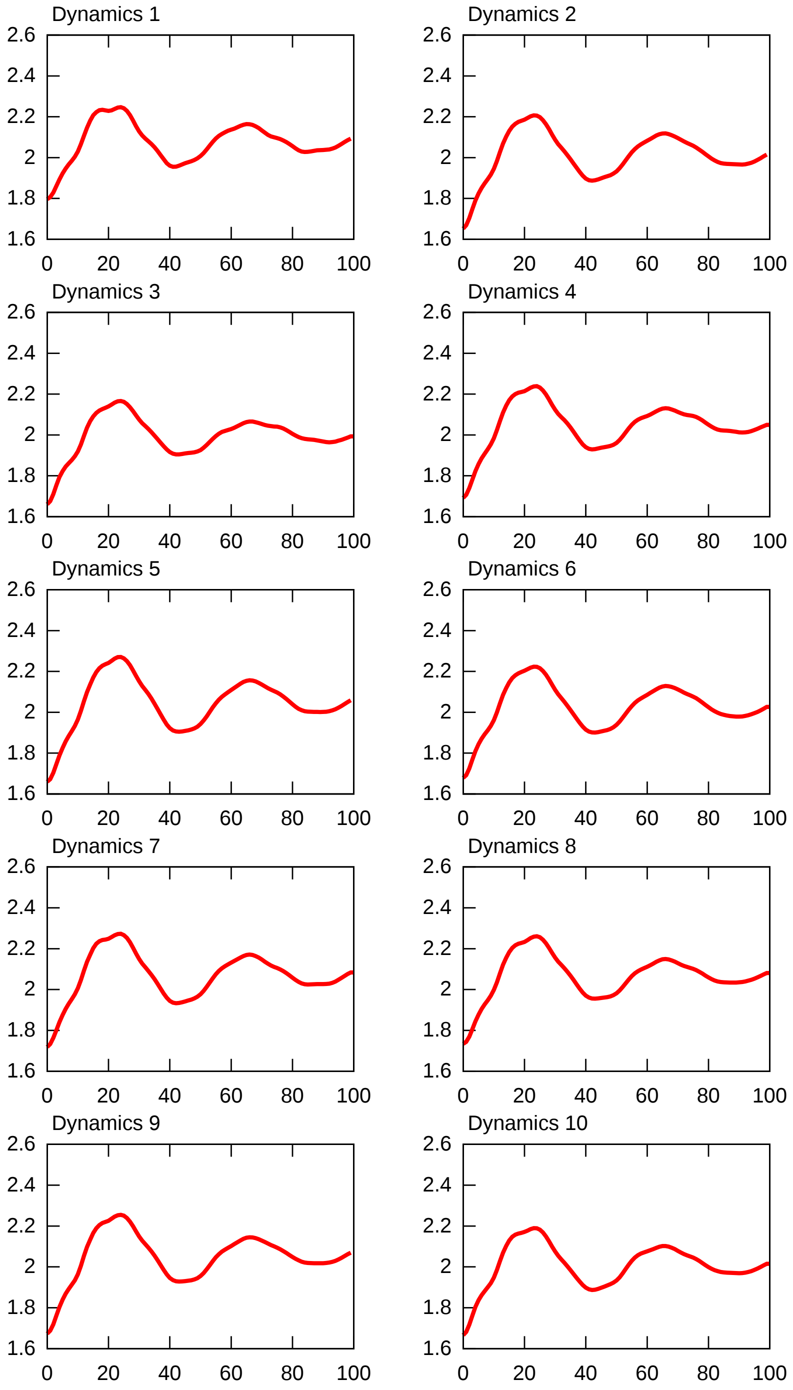


**Supplementary Figure 8. Singlet manifold evolution of dissociation coordinate.** Evolution of the Fe-C(O) distance (in Ångstrom) for the first 0.1 ps, using the different initial conditions specified in Supplementary Table 8.

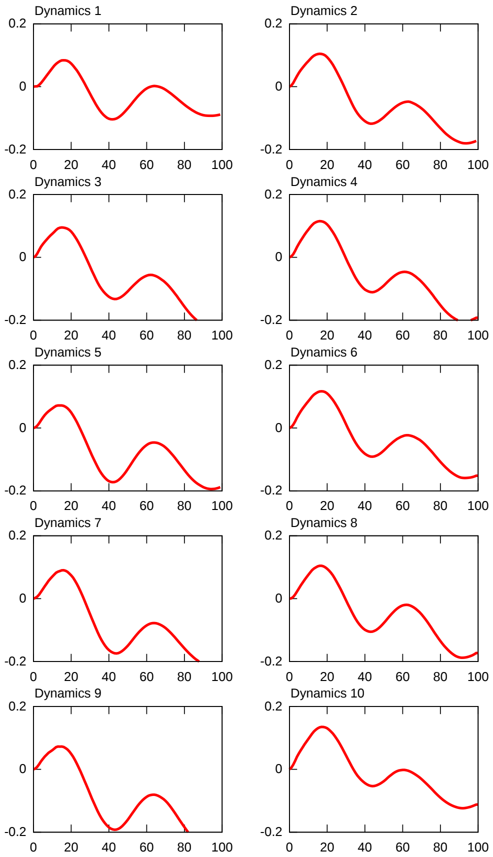


**Supplementary Figure 9. Singlet manifold evolution of the Fe OOP coordinate.** Dynamic evolution of the Fe out-of-plane distance for the singlet only model during the first 0.1 ps. The distance is calculated as the difference between the Fe z-coordinate position and the rest z-coordinate position (corresponding to the minimum energy geometry optimized at the B3LYP/LANL2DZ level). Positive and negative values of Fe indicates that Fe is going towards and contrary to the CO position respectively.

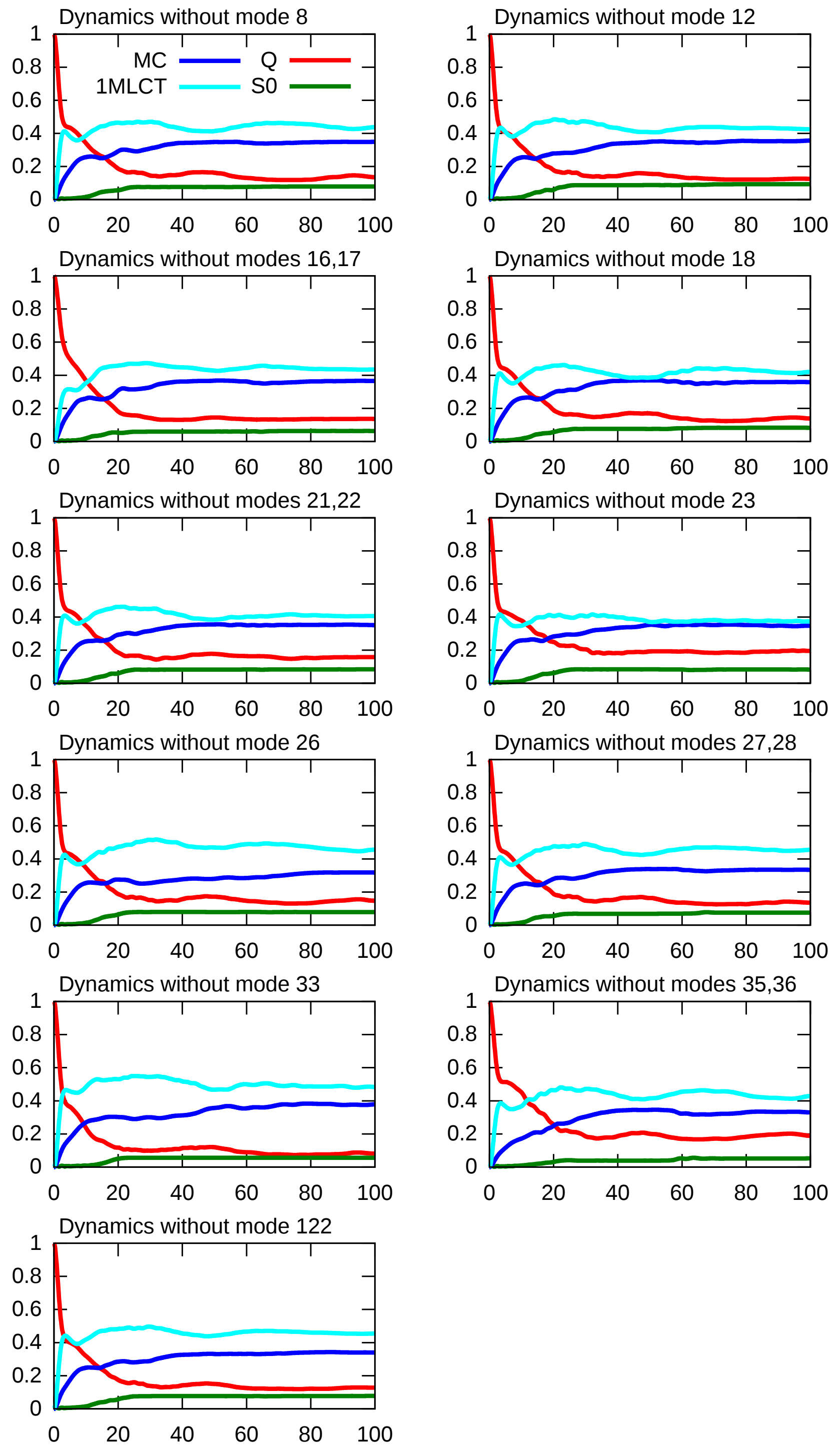


**Supplementary Figure 10. Population evolution excluding normal modes.** Effect of each mode on the population evolution on the singlet model. In each dynamics, we exclude the take out one mode (for singly-degenerate symmetries) or two modes (for doubly-degenerate symmetries) from the singlet model. For details of the modes, see Supplementary Table 10. The initial conditions are as in dynamics 1 of Supplementary Table 8.

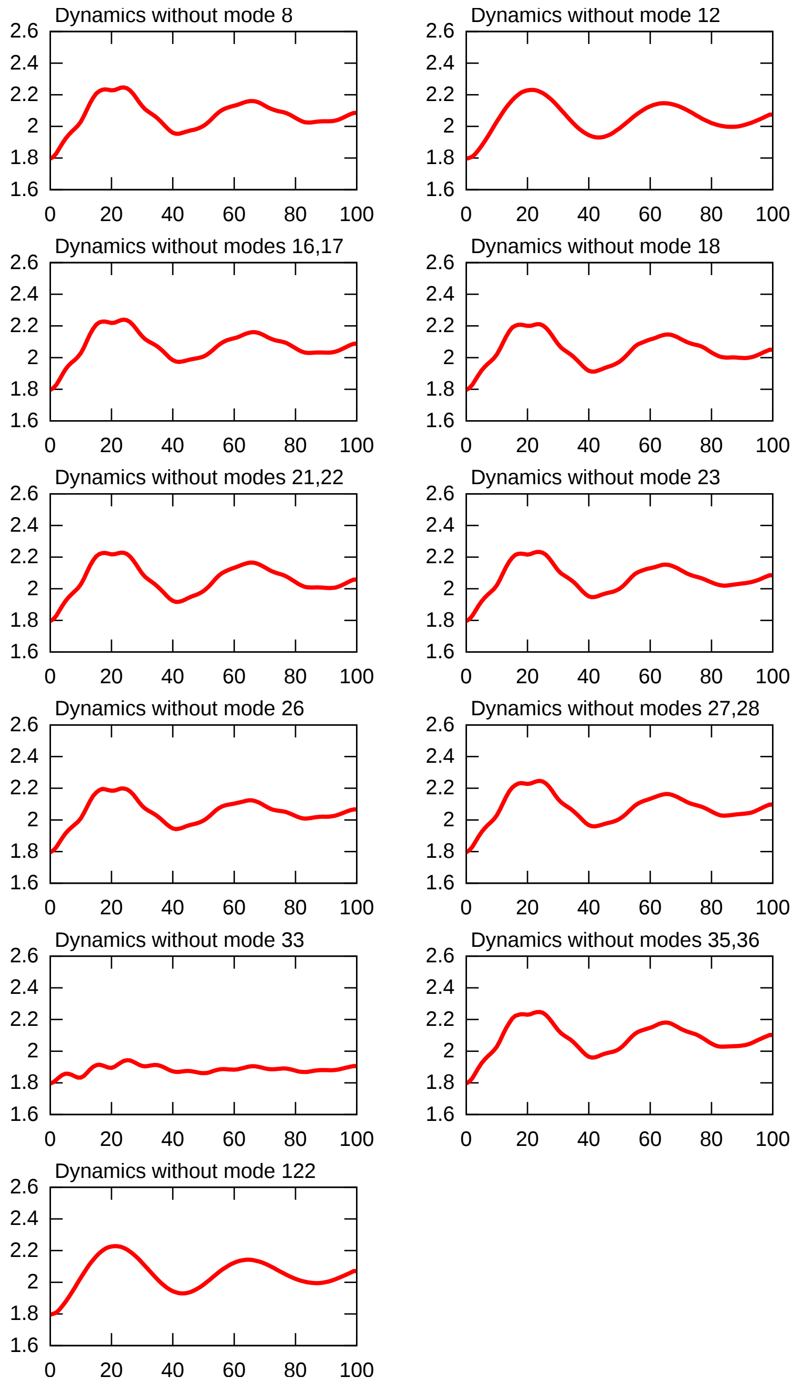


**Supplementary Figure 11. Evolution of dissociation coordinate excluding certain normal modes.** Evolution of the Fe-C(O) distance (in Ångstrom) for the first 0.1 ps, excluding normal modes from the dynamics (see caption of Supplementary Figure 10 for details).

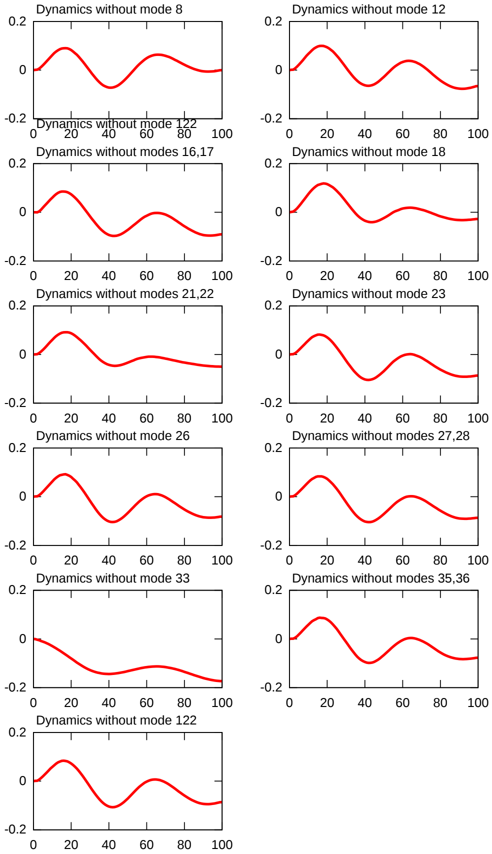


**Supplementary Figure 12. Evolution of the Fe OOP coordinate excluding normal modes.** Dynamic evolution of the Fe out-of-plane distance for the singlet only model during the first 0.1 ps excluding normal modes (see caption of Supplementary Figure 10 for details). The distance is calculated as the difference between the Fe z-coordinate position and the rest z-coordinate position (corresponding to the minimum energy geometry optimized at the B3LYP/LANL2DZ level). Positive and negative values of Fe indicates that Fe is going towards and contrary to the CO position respectively.

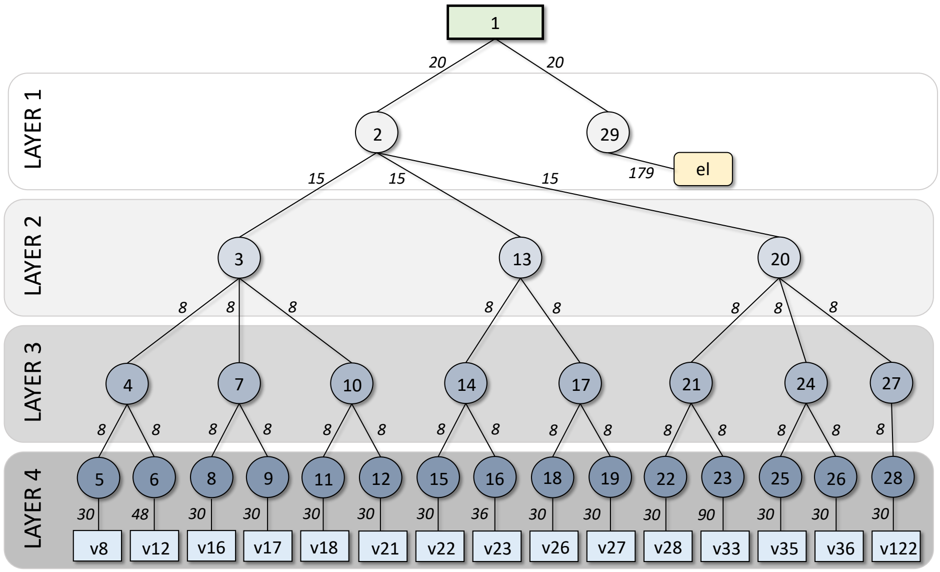


**Supplementary Figure 13. Layer structure of the ML-MCTDH wavefunction.** Schematic representation of the ML-MCTDH multi-layer tree structure where 179 electronic states and 15 vibrational modes are arranged into 4 layers.

**Supplementary Note 1: Timescales.** Due to the large number of electronic states, it is difficult to define a precise kinetic model involving each individual state. For extracting kinetic constants to be compared with experimental results, we have rather considered the following kinetic reactions:

- For the full quantum dynamics with summed up populations according to the spin multiplicity, the reaction $^1\mathrm{S}\xrightarrow{k_{S-T}}{}^3\mathrm{T}\xrightarrow{k_{T-Q}}{}^5\mathrm{Q}$ allows us to extract the kinetic constants corresponding to the total singlet-triplet ($k_{S-T}$) and the total triplet-quintet ($k_{T-Q}$) population transitions.

- For the full quantum dynamics with separated populations according to the state nature, the reaction $^1\mathrm{Q}\xrightarrow{k_Q}{}^1\mathrm{MLCT}$ allows us to extract in addition to $k_Q$ the triplet-quintet MLCT time scale.

The fitting model for $\tau_Q = 1/k_Q$ and $\tau_{S-T} = 1/k_{S-T}$ is $\mathrm{e}^{-k_Q t}$ and $\mathrm{e}^{-k_{S-T} t}$ respectively. For $\tau_{T-Q} = 1/k_{T-Q}$ the model is 1-$\mathrm{e}^{-k_{T-Q} t}$. In Supplementary Table 9, we summarize the kinetic contants for the 10 dynamical models. The average values obtained and the standard deviation are $\tau_Q = 25.73 \pm 7.05$, $\tau_{S-T}$=76.25 $\pm$ 15.92 and $\tau_{T-Q}$=429.18 $\pm$ 70.06.

**Supplementary Note 2: Electronic structure.** All geometries have been optimized at the density functional theory (DFT) level using the LAN2DZ basis set,[2] which includes pseudopotentials to treat the relativistic effects of Fe. For such calculations, the GAUSSIAN09 software has been employed.[3] We have employed B3LYP functional for all calculations.[4–6] Tests on the B3LYP* functional (same parameters as B3LYP but employing 25% of Hartree-Fock exchange), which was reported to reproduce better the low-spin to high-spin transition was tested, but the results were not improved with respect to B3LYP.[7] It is for this reason, that the electronic structure calculations for the excited states has been performed at the CASSCF/CASPT2 level of theory.[8,9] Multi-reference calculations have been performed with Molcas 8.[10] No symmetry has been employed in the geometry optimizations, although the structures in the singlet ground state minimum show a quasi-$C_{4v}$ symmetry. All minimum energy structures have been characterized by a frequency calculation. All CASSCF calculations have been done using 10 electrons in 9 orbitals. The orbitals are depicted in Supplementary Figure 1. In our model, we only include four orbitals for the porphyrin and five d orbitals. Since we consider the model for excitation to the Q band, we do not need to include further states for the porphyrin, and so the B band, which is located 1 ev higher than the Q band, is excluded from the system. We have selected a space of 19 singlets, 20 triplets and 20 quintets, which converge the continuum region around the Q-band. Relativistic effects have been included using the two-component Douglas-Kross-Hess Hamiltonian (DKH).[11,12] For calculating the potential energy surface, the ANO-RCC-VDZ basis set has been employed.[13,14] The effects of the basis set size are negligible (see further Supplementary Table 1). The absorption spectrum for the Imidazole-Heme-CO model, the Heme-CO model and the Imidazole-Heme can be found in Supplementary Tables 3, 4 and 5 respectively. We observe in all cases that a continuum band appears above the metal-centred states in all cases. This band is represented by configuration state functions that correspond to double (for singlet and triplet) and triple (for quintet) excitations with respect to the reference configuration. This implies that only multiconfiguration methods to the third order can be used for representing correctly the static correlation effects of the complex. Therefore, the interpretation of heme-CO complex based on the TDDFT results reported previously in the literature cannot be considered correct. The CASPT2 calculations have been performed using the multi-state approach, with an imaginary shift of 0.01 au to avoid intruder state problems. The MS-CASPT2 calculation of the potential energy surfaces has been performed freezing 50 virtual states, which accelerate the computations. Still, the spin-crossover of the ground state is qualitatively well represented. Furthermore, the minimum energy structures at the DFT level are also minima of the MS-CASPT2 protocol. The QM/MM calculations have been performed with the Tinker/Gaussian09 and Tinker/Molcas interface employing the ElectroStatic Potential Fitting method.[15] We have employed the CHARM22 force field with CMAP corrections.[16] The protein structure has been taken as the mean value X-ray structure complexed with CO of *Equus caballus* (RCSB PDB code: 5CN5).[1] The QM region comprises the full heme, the CO and the proximal histidine. A link atom has been used to cut the $C_\alpha$-$C_\beta$ bond. We have optimized the QM region with frozen MM region at the B3LYP/LANL2DZ level of theory. Only the water molecules appearing in the 5CN5 structure where kept and no extra water molecules were added. The electronic structure calculations have been performed with CASSCF/CASPT2/ano-rcc-vdz. Due to the computational cost, and for the sake of comparison, we have only performed the calculations over the 12 lowest roots.

**Supplementary Note 3: Anharmonic couplings.** In the harmonic approximation, the vibrational potential has the following form (in mass-frequency-weighted Cartesian coordinates):

$$V(q_i) = \sum_i \frac{1}{2}\omega q_i^2 \,, \tag{1}$$

Anharmonic couplings between vibrational modes can induce mode mixing, which is responsible of vibrational energy redistribution:

$$V(q_i) = \sum_i \frac{1}{2}\omega_i q_i^2 + \sum_{ijk} V_{i,j,k} q_i q_j q_k \,. \tag{2}$$

These equations are valid for the ground-state only. In the excited states, Duschinsky rotations should be included to account for the mixing of normal modes.[17] In Supplementary Table 11, we show the computed anharmonic couplings using GAUSSIAN09.[3] Remarkably, the dissociative mode (mode 33) is coupled to the doming mode (mode 26). As we have shown, the doming is activated after photolysis, and it serves to relax the structure of the high-spin state. A very strong coupling exist also between the doming mode (mode 26) with an in-plane distortion (mode 22) involving a rotation of CO. However, these modes are not directly involved in the dissociation process, and therefore it is justified to exclude them from the model. Anharmonic couplings, especially couplings to vibrational modes of the protein, will become important at times larger than 500 fs as shown experimentally.[1].

**Supplementary Note 4: Quantum dynamics.** Quantum dynamical calculations were carried out for the vibronic coupling Hamiltonian specified in section "Methods" of the main text, for 179 electronic states and 15 vibrational modes, using the Multi-Layer Multi-Configuration Time-Dependent Hartree (ML-MCTDH) method.[18–22] The ML-MCTDH method is a hierarchical tensor scheme which defines the wavefunction in terms of a multi-layer tree structure consisting of combinations of single-particle functions (spf's). The tree structure employed in the present calculations is illustrated in Supplementary Figure (13). Here, vertices indicate spf's (with an arbitrary numbering scheme) while edges give information on the number of spf's. Four layers were used, with the number of spf's varying between $n_{\rm spf} = 8$ and $n_{\rm spf} = 20$. All electronic states are grouped together in a formal electronic spf in the first layer. In the last layer, each one-dimensional spf is represented explicitly by a harmonic-oscillator (HO) Discrete Variable Representation (DVR) with up to 90 DVR points. The ML-MCTDH method has a unique capacity to treat the highly correlated vibronic dynamics that characterizes the present system. While full convergence is difficult to achieve, the present set of calculations are well converged in the sense that only small variations in the dynamics are observed when modifying the number of spf's.

## SUPPLEMENTARY REFERENCES


[1] Barends, T. R. *et al.* Direct observation of ultrafast collective motions in CO myoglobin upon ligand dissociation. *Science* **350**, 445–450 (2015).

[2] Hay, P. J. & Wadt, W. R. Ab initio effective core potentials for molecular calculations. potentials for k to au including the outermost core orbitals. *J. Chem. Phys.* **82**, 299 (1985).

[3] Gaussian 09, Revision D.01, M. J. Frisch, G. W. Trucks, H. B. Schlegel, G. E. Scuseria, M. A. Robb, J. R. Cheeseman, G. Scalmani, V. Barone, G. A. Petersson, H. Nakatsuji, X. Li, M. Caricato, A. Marenich, J. Bloino, B. G. Janesko, R. Gomperts, B. Mennucci, H. P. Hratchian, J. V. Ortiz, A. F. Izmaylov, J. L. Sonnenberg, D. Williams-Young, F. Ding, F. Lipparini, F. Egidi, J. Goings, B. Peng, A. Petrone, T. Henderson, D. Ranasinghe, V. G. Zakrzewski, J. Gao, N. Rega, G. Zheng, W. Liang, M. Hada, M. Ehara, K. Toyota, R. Fukuda, J. Hasegawa, M. Ishida, T. Nakajima, Y. Honda, O. Kitao, H. Nakai, T. Vreven, K. Throssell, J. A. Montgomery, Jr., J. E. Peralta, F. Ogliaro, M. Bearpark, J. J. Heyd, E. Brothers, K. N. Kudin, V. N. Staroverov, T. Keith, R. Kobayashi, J. Normand, K. Raghavachari, A. Rendell, J. C. Burant, S. S. Iyengar, J. Tomasi, M. Cossi, J. M. Millam, M. Klene, C. Adamo, R. Cammi, J. W. Ochterski, R. L. Martin, K. Morokuma, O. Farkas, J. B. Foresman, and D. J. Fox, Gaussian, Inc., Wallingford CT, 2016.

[4] Becke, A. D. Density–functional exchange–energy approximation with correct asymptotic behavior. *Phys. Rev. A* **38**, 3098 (1988).

[5] Lee, C., Yang, W. & Parr, R. G. Development of the colle–salvetti correlation–energy formula into a functional of the electron density. *Phys. Rev. B* **37**, 785 (1988).

[6] Vosko, S. H., Wilk, L. & Nusair, M. Accurate spin–dependent electron liquid correlation energies for local spin density calculations: a critical analysis. *Can. J. Phys.* **58**, 1200–1211 (1980).

[7] Reiher, M., Salomon, O. & Hess, B. A. Reparameterization of hybrid functionals based on energy differences of states of different multiplicity. *Theor. Chem. Acc.* **107**, 48–55 (2001).

[8] Roos, B. J. *The Complete Active Space Self–Consistent Field Method and its Applications in Electronic Structure Calculations.*, vol. 69 of *Adv. Chem. Phys.*, 399 (John Wiley and Sons, Ltd., 1987).

[9] Andersson, K., Malmqvist, P. & Roos, B. O. Second–order perturbation theory with a complete active space self–consistent field reference function. *J. Chem. Phys.* **96**, 1218–1226 (1992).

[10] Aquilante, F. *et al.* Molcas 8: New capabilities for multiconfigurational quantum chemical calculations across the periodic table. *J. Comput. Chem.* **37**, 506–541 (2016).

[11] Douglas, M. & Kroll, N. M. Quantum electrodynamical corrections to the fine structure of helium. *An. Phys.* **82**, 89–155 (1974).

[12] Hess, B. A. Relativistic electronic-structure calculations employing a two-component no-pair formalism with external-field projection operators. *Phys. Rev. A* **33**, 3742–3748 (1986).

[13] Roos, B. O., Lindh, R., Malmqvist, P. A., Veryazov, V. & Widmark, P. O. Main group atoms and dimers studied with a new relativistic ano basis set. *J. Phys. Chem. A* **108**, 2851 (2005).

[14] Roos, B. O., Lindh, R., Malmqvist, P. A., Veryazvov, V. & Widmark, P. O. New relativistic ano basis sets for transition metal atoms. *J. Phys. Chem. A* **109**, 6575–6579 (2005).

[15] Melaccio, F., Olivucci, M., Lindh, R. & Ferré, N. Unique qm/mm potential energy surface exploration using microiterations. *Int. J. Quant. Chem.* **111**, 3339–3346 (2011).

[16] Mackerell, A. D. Empirical force fields for biological macromolecules: Overview and issues. *J. Comput. Chem.* **25**, 1584–1604 (2004).

[17] Olbrich, G. & Kupka, H. The Duschinsky effect and optical spectra. *Z. Naturforsch.* **38a**, 937–946 (1983).

[18] G. A. Worth, M. H. Beck, A. Jäckle, O. Vendrell, and H.-D. Meyer, The MCTDH Package, Version 8.5.5., see http://mctdh-uni-hd.de.

[19] Wang, H. & Thoss, M. Multilayer formulation of the multiconfiguration time–dependent hartree theory. *J. Chem. Phys.* **119**, 1289 (2003).

[20] Meyer, H.-D., Manthe, U. & Cederbaum, L. S. The multi–configurational time–dependent hartree approach. *Chem. Phys. Lett.* **165**, 73–78 (1990).

[21] Beck, M., Jäckle, A., Worth, G. & Meyer, H.-D. The multiconfiguration time-dependent Hartree (MCTDH) method: a highly efficient algorithm for propagating wavepackets. *Phys. Rep.* **324**, 1–105 (2000).

[22] Vendrell, O. & Meyer, H.-D. Multilayer multiconfiguration time-dependent Hartree method: Implementation and applications to a Henon-Heiles Hamiltonian and to pyrazine. *J. Chem. Phys.* **134**, 044135 (2011).
